# The Exposome and the Kidney: A Silent Dialogue Shaping Chronic Kidney Disease

**DOI:** 10.3390/jox15030073

**Published:** 2025-05-14

**Authors:** Livia Alvarenga, Marcia Ribeiro, Ludmila F. M. F. Cardozo, Natália A. Borges, Peter Stenvinkel, Denise Mafra

**Affiliations:** 1 Department of Cardiopneumology, Faculty of Medicine, University of São Paulo (FMUSP), São Paulo 05403-903, Brazil; liviaalvarenga@id.uff.br; 2 Graduate Program in Nutrition Science, Federal Fluminense University, Niterói 24220-900, Brazil; ludmilacardozo@id.uff.br; 3 Graduate Program in Biological Sciences—Physiology, Federal University of Rio de Janeiro (UFRJ), Rio de Janeiro 21941-901, Brazil; marciaribeiro@biof.ufrj.br; 4 Graduate Program in Cardiovascular Sciences, Federal Fluminense University, Niterói 24220-900, Brazil; 5 Graduate Program in Food, Nutrition and Health—Institute of Nutrition, State University of Rio de Janeiro (UERJ), Rio de Janeiro 20950-000, Brazil; natalia.borges@uerj.br; 6 Division of Renal Medicine and Baxter Novum, Department of Clinical Science, Technology and Intervention, Karolinska Institutet, 171 77 Stockholm, Sweden; peter.stenvinkel@ki.se

**Keywords:** exposome, general external exposome, specific external exposome, pollution, global warming, diet, chronic kidney disease

## Abstract

Genetic predisposition accounts for less than 20% of the global disease burden, highlighting the substantial role of environmental factors in health outcomes. In chronic kidney disease (CKD), a growing global prevalence, understanding the interplay between genes and the environment is crucial. Emerging research in the exposome and genome underscores how environmental exposures interact with genetic variants to influence the development and progression of CKD. The term “exposome” encompasses a variety of factors, including personal behaviors like smoking, a sedentary lifestyle, and making specific dietary choices (such as consuming ultra-processed foods, sugar, or fat). It also includes broader determinants such as pesticides, air, water, and soil pollution, nanoplastics, global warming, stressful life events, and socioeconomic status. Research on the exposome significantly increases our understanding of toxicological processes and individual variations in susceptibility to environmental stressors. This narrative review aims to explore the exposome associated with CKD, highlight key environmental exposures in its development, and discuss potential preventive and therapeutic strategies informed by these exposure-related factors.

## 1. Introduction

Since vital manifestations depend on external physical and chemical conditions, the notion of an internal vital principle unaffected by these external influences becomes untenable [1]. The lifelong accumulation of environmental exposures and their physiological effects on the body is called the exposome. These effects include metabolic changes, protein modifications, DNA mutations, epigenetic modifications, and alterations in the intestinal microbiome [2].

While mapping the human genome has significantly advanced our ability to explore the genetic underpinnings of diseases, its predictive power for many common diseases remains limited. For numerous complex human diseases, such as cancer, cardiovascular disease (CVD), respiratory disease, and type-2 diabetes, genetic variation accounted for a minor part of the population attributable fraction associated with genetic predisposition [3]. Thus, integrating exposome research with genomic studies has proven effective in various fields, such as nephrology, where genetic variations associated with significant environmental factors contributing to chronic kidney disease (CKD) have been identified [4,5,6].

The kidneys are crucial in maintaining water and electrolyte balance, removing toxins, and protecting us from environmental stressors. As essential filters for environmental contaminants, they are particularly vulnerable to damage in polluted, dry, and hot conditions [7]. This narrative review discusses the general and specific external exposome related to CKD, emphasizing environmental exposures associated with its development. It also provides insights into potential preventive and therapeutic approaches based on exposure factors.

## 2. The Role of Exposomes in Kidney Health

A new line of research involving kidney disease and the interaction between the external environment is emerging. The cumulative and interactive effects of multiple exposures, including chemical, physical, biological, and social factors, may aggravate the risk of CKD.

More advanced techniques are currently available in this context, utilizing high-resolution mass spectrometry, a powerful instrument capable of detecting thousands of tiny molecules in biological materials such as plasma and urine. High-resolution mass spectrometry can identify numerous drugs and environmental chemicals’ metabolites [8]. Additionally, a combination of mass spectrometry and chromatographic separation is typically employed, with liquid chromatography being the most effective for detecting a wide range of molecules, particularly water-soluble polar metabolites, while gas chromatography is the most efficient for identifying nonpolar, volatile, and lipophilic compounds [9]. Thus, new compounds can be identified through mass spectral fragmentation, similar to proteomic methodologies in which molecules undergo fragmentation, and the resulting components are identified and reassembled through bioinformatics processes [10].

Furthermore, it is worth highlighting that geographic location is a valuable piece of data in studies, as geographic uncertainties can obscure associations between environment and health or generate spurious risks, leading to contextual uncertainties [11]. The incidence of CKD resulting from infections and unknown causes in specific geographic locations worldwide continues to challenge healthcare research [12,13]. Thus, scientific and technological advances such as geographic information systems, remote sensing, global positioning systems, geolocation technologies, and portable and personal sensing are essential for improving the assessment of exposure variability and the analytical methods themselves [14].

**Figure 1:**
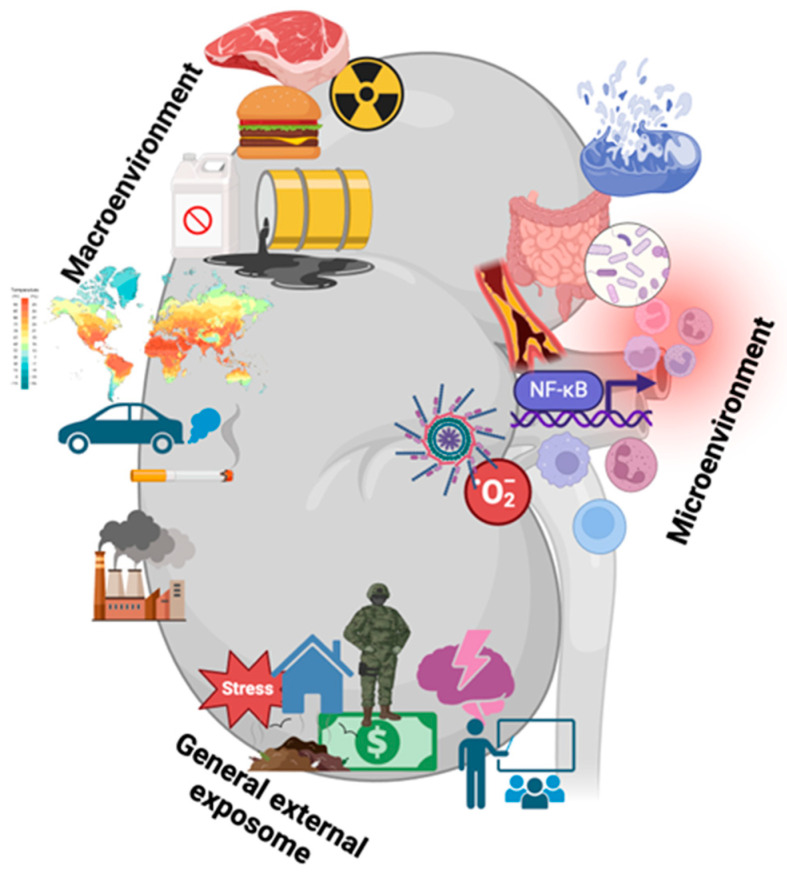
Exposome factors that may aggravate CKD progression. The exposome includes the microenvironment (gut microbiota, inflammation, oxidative stress, and senescence), the macroenvironment (radiation, pollution, unhealthy diet, tobacco, and global warming), and the general external exposome (war, poverty, stress, cultural, economic, and social capital). Created by Biorender.com.

The exposome can be divided into three main domains: the general external exposome, which encompasses the physical space in which one lives; the social environment, referring to the social relationships and contexts in which groups of people coexist; the physicochemical environment, consisting of chemical or physical agents present in our local area; and the lifestyle and food environment, concerning the accessibility, availability, and affordability of food in the neighborhood. The specific external exposome includes individual-level exposures, such as health behaviors, financial/income situations, and pollution exposure. Lastly, the internal exposome is characterized by internal biological processes, including metabolism and the microbiome, which can be influenced by external exposures [15]. Figure 1 illustrates these interactions related to kidney health.

### 2.1. General External Exposome

#### 2.1.1. Global Warming

Heat waves have increased significantly due to the rise in global temperature, increasing the risk of illness and fatality. The kidneys become essential for heat-associated conditions, protecting the body from heat and dehydration [16]. High temperatures can increase blood hyperosmolality, dehydration, and elevated core body temperatures, which may accelerate the onset of CKD. Indeed, this is particularly concerning in hot climates, where workers are exposed to extreme heat, raising the alarm about a potential epidemic of CKD among those affected [17]. One study examined data on hospital admissions over 15 years, with the estimated risk of hospitalization for kidney disease within 0 to 7 days increasing by 0.9% for each 1 °C increase in average daily temperature. This risk was higher in women, children, and people > 80 years. As a result, rising temperatures may be to blame for 7.4% of renal disease hospitalizations [18].

During heat stress, dehydration is commonly observed because fluids often fail to adequately replace the significant amount of water lost through sweating. Since sweat is hypoosmotic relative to plasma, losing body water results in intracellular dehydration, affecting intra- and extracellular fluid compartments. Consequently, this leads to hyperosmolality and hypovolemia [19]. This increase in serum osmolality can trigger vasopressin secretion and the activation of the aldose reductase pathway, leading to kidney injury [20].

Historically, dehydration has been associated with acute kidney injury (AKI) and reversible features [21]. However, a recent epidemic of CKD among manual laborers in Central America suggests that recurrent dehydration linked to heat is a predominant risk factor [22]. A systematic review and meta-analysis reported that 15% of individuals who regularly worked under heat stress developed CKD or AKI [23]. Exposure to heat can be one of the causes of kidney changes and can also worsen kidney function in already-diagnosed patients. A post hoc analysis of the DAPA-CKD study revealed a link between exposure to ambient heat and an accelerated decline in the estimated glomerular filtration rate (eGFR) (3.7 mL/min/1.73 m^2^ per year) [24].

In addition, heat stress can initiate the inflammatory pathway, specifically via transcription factor nuclear factor-kappa B (NF-κB), which is an essential modulator of the inflammatory response [25]. Thermal stress can also trigger cellular senescence, which accelerates aging and contributes to the development of age-related diseases [26]. With temperatures worldwide rising, it is imperative to investigate how heat stress leads to kidney illness, develop efficient hydration plans, and lessen the adverse effects of extended heat exposure [27].

#### 2.1.2. Air Pollution

Air pollution results from a complex mixture of gaseous components and solid and liquid particles in the atmosphere, primarily emanating from the combustion of coal, gasoline, and diesel fuel [28]. The main components of atmospheric pollution are gaseous compounds, nitrogen dioxide, carbon monoxide (from road traffic and the burning of industrial fuels), ozone (O_3_), and sulfur oxides (from industries) [29].

These gases alter gene expression in antioxidant response pathways, inflammatory signaling, and kidney endothelial dysfunction. They decrease renal blood flow and increase oxidative stress and inflammation, which can damage DNA [30,31].

O_3_ is a primary oxidant in photochemical pollution, and studies have shown that O_3_ concentrations are negatively associated with eGFR [32]. The proposed mechanism for the association between O_3_ exposure and CKD remains obscure, but O_3_ exposure could increase fasting plasma glucose and alter the lipid profile, contributing to increased insulin resistance. Also, O_3_ induces oxidative and endoplasmic reticulum stress, contributing to the development of risk comorbidities and CKD through tubulointerstitial damage and fibrosis of renal tissue [32,33].

Particulate matter (PM) is a significant component of air pollution and a mixture of tiny solid particles and liquid droplets. It can be PM10 (with a diameter of 10 μm or less) or PM2.5 (2.5 μm or less). According to the Global Burden of Disease study, approximately 5 million annual deaths are caused by PM2.5, which activates inflammatory cells and disrupts the integrity of lung epithelial cells, leading to neutrophil recruitment, mitochondrial dysfunction, reactive oxygen species (ROS) production [34], inflammasome activation, and increased production of inflammatory cytokines [35].

A meta-analysis of 7,967,388 participants, including cohort and cross-sectional studies until 2023, revealed that each 10 μg/m^3^ increase in airborne PM2.5 is associated with increased CKD incidence and prevalence. Furthermore, the association of PM2.5 exposure with the incidence of end-stage kidney disease (ESKD) suggests an increased risk when the duration of exposure extends beyond ten years [36]. A cohort study with 47,204 adults demonstrated that a 10 μg/m^3^ increase in PM1 (air particles with a diameter ≤ 1 μm) was associated with an increased risk of CKD and albuminuria. These findings highlight the critical need for implementing air pollution control strategies to alleviate the burden of CKD [37].

#### 2.1.3. Water and Soil Pollution

People exposed to plastic-derived substances in water, such as phthalates and bisphenol A, may experience endocrine disruption and immunotoxicity, potentially causing nephrotoxic effects [38]. Furthermore, nanoplastics can lead to the destruction of renal tissue structure and function through ferroptosis. As nanoplastics enhance the binding of serum transferrin to its receptor on the cell membrane, this increases intracellular iron levels and elevates oxidative stress and lipid peroxidation, making cells more susceptible to ferroptosis [39].

Moreover, toxic heavy metals can increase the generation of ROS in the body, contributing to oxidative stress and cellular changes in kidney damage [40]. Several heavy metals, such as arsenic, cadmium, lead, mercury, and uranium, displace parent ions from protein locations in renal tubular cells; these toxic heavy metals can operate as nephrotoxic agents, interfering with the normal physiological functioning of renal tubular cells [40].

Exposure to arsenic, lead, and cadmium can affect the expression of metalloproteinase 3 (TIMP3) tissue inhibitors related to hypertension, CVD, and renal health fibrosis. According to a case-control study, the odds ratio of CKD in people with the TIMP3rs9609643 GA AA genotype is higher than in people with the GG genotype, especially with elevated levels of lead in blood and total arsenic in urine. Furthermore, the study found that the TIMP3rs9609643 GG genotype and high levels of lead in the blood tend to interact, increasing the incidence of CKD [41].

Furthermore, cadmium toxicity is linked to the dysregulation of zinc and copper homeostasis, causing the displacement of these minerals, which results in a decrease in their antioxidant enzymes and an increase in the cytoplasm, ultimately leading to changes in the redox state of the organism [42]. Thus, conformational changes and the inhibition of enzymatic activity, such as cytoplasmic superoxide dismutase (Cu/Zn-SOD), are observed, leading to an increased production of ROS by the Fenton reaction [42]. A study investigated whether cadmium exposure impairs renal metal reabsorption in 200 individuals. It was observed that higher cadmium exposure is associated with lower levels of zinc reabsorption and higher serum copper/zinc ratios, which correlate with greater tubular impairment [43]. The serum copper–zinc ratio seems strongly associated with urinary levels of beta-2 microglobulin and malondialdehyde and the risk of renal tubular injury linked to urinary cadmium levels [44].

Another environmental heavy metal known to induce nephrotoxic effects, such as glomerular and tubular lesions, is mercury (Hg). After exposure, mainly from ingesting contaminated fish, Hg is readily absorbed by the gastrointestinal tract and kidneys, in which the renal proximal tubule is the leading site of capture and accumulation of mercuric species [45]. Hg ions bound to biomolecules like selenium or thiol-containing compounds (e.g., GSH, cysteine, homocysteine, N-acetylcysteine, or albumin) enter renal tubules as conjugates, crossing the luminal membrane through amino acid transporters and the basolateral membrane via organic anion transporters. Upon entering the cell, mercuric conjugates cause changes in DNA methylation and repair, inflammation, mitochondrial damage, and oxidative stress [46].

Also, exposure to lead and copper can lead to proteinuria and a reduction in the eGFR, confirming heavy metals’ contribution to CKD development [47]. In corroboration with this, it has been reported that patients were more likely to develop CKD due to exposure to these heavy metals [48]. A study of 45,000 participants from Taiwan observed a high prevalence of CKD related to arsenic contamination in groundwater [49].

Additionally, organic solvents such as trichloroethylene (TCE), perchloroethylene (PCE), and other solvents used in manufacturing and cleaning can contaminate soil and groundwater due to improper disposal. Prolonged exposure can cause kidney damage [50]. Common mechanisms of action for TCE and PCE include the glutathione (GSH) conjugation pathway, which produces nephrotoxic metabolites, and the cytochrome P450 (CYP)-dependent oxidation pathway [51].

Furthermore, reactive metabolites derived from S-cysteine conjugates of organic solvents cause thiol oxidation, oxidative stress, mitochondrial dysfunction, and protein and DNA alkylation. Acute exposure has been associated with changes in gene expression and cell death. In contrast, chronic exposure—even at low doses—has been associated with kidney cancer due to both genotoxic and nongenotoxic changes [51].

Moreover, studies have shown a positive association between the use of pesticides, such as hexachlorocyclohexane (HCH), endosulfan, alachlor, and pendimethalin, and impaired renal function [52,53]. The primary surfactant herbicide associated with the development of CKD is glyphosate surfactant herbicide (GPSH) that uncouples mitochondrial oxidative phosphorylation [54] and causes nephrotoxicity through the activation of N-methyl-D-aspartate receptors (NMDARs), central receptors of the glutamatergic system expressed in the proximal tubular epithelium [55]. The activation of NMDARs results in the opening of a non-selective ion channel for cations, increasing cytoplasmic calcium ions (Ca^2+^) and oxidative damage [56].

Therefore, effective planning and management of water resource systems, focusing on human health, are crucial for mitigating risk factors associated with water exposure and CKD [57].

#### 2.1.4. Socioeconomic Factors

Social and environmental exposures vary across geographic and socioeconomic contexts. In high-income countries, air pollution, a sedentary lifestyle, and dietary excess may be predominant risk factors for CKD, while in low- and middle-income settings, limited access to clean water, nutrient deficiencies, and exposure to infectious agents may represent more significant concerns [58,59]. Additionally, aging is universally linked to the risk of chronic diseases, and this interaction with environmental factors such as solar radiation, particulate matter, and poor nutrition may be more pronounced in socially and economically deprived community populations [60,61]. These disparities highlight the importance of adopting a geographically contextualized approach when investigating the role of the exposome in CKD development and progression [62]. Public health strategies must consider local realities to ensure more effective and equitable outcomes interventions [63].

The location of a population’s housing, along with income, education, and economic investment in the neighborhood, can influence food choices and dietary patterns, thereby affecting the development of diseases [64]. In this context, the presence or absence of supermarkets may determine how the built environment affects access to healthy and nutritious food [65]. Communities that live farther away from supermarkets, supercenters, or large grocery stores often have lower income and education levels while having greater access to independent grocery stores and convenience outlets. Unlike supermarkets, grocery stores typically charge higher prices, offer fewer fruits and vegetables, and stock more energy-dense food options [66]. Thus, communities facing resource and nutrition deprivation may elevate the risk of obesity, cardiovascular disease, and CKD [65].

The association between CKD and low socioeconomic status is noteworthy [67]. Consequently, more comprehensive screening methods must consider patients’ socioeconomic circumstances and medical conditions such as diabetes and hypertension [68], providing valuable information to better manage and avoid CKD [69]. Poor socioeconomic status is a critical factor in the relationship with CKD, and there are regional and population differences in the prevalence of CKD. This discrepancy can be significantly influenced by cultural factors, dietary habits, environmental aspects like air pollution, and healthcare access [64].

Furthermore, access to healthcare, specifically the availability of quality healthcare services, refers to those achieving the best possible outcomes in care, problem resolution, securing all necessary materials for quality care, and welcoming the population. This can significantly influence the development of CKD [70]. Since limited access to healthcare can lead to untreated hypertension, diabetes, and other conditions that result in CKD. According to Grant et al., patients with CKD in stages 1–5 who are not on dialysis and who are socioeconomically disadvantaged experience faster disease progression and have an increased risk of premature death from cardiovascular diseases. Despite this, the author emphasizes that consolidating the contribution of socioeconomic factors in CKD is challenging due to the interdependent nature of the social determinants of health [71].

### 2.2. Specific External Exposome

#### 2.2.1. Unhealthy Diet

Diet constitutes a significant domain within exposome research, establishing a robust connection between the prevention and occurrence of numerous non-communicable diseases, including CKD [72,73]. Currently, there is a worrying increase in the consumption of ultra-processed foods (UPFs), characterized by high levels of sodium, saturated fats, phosphate, and sugar, which is observed, exacerbating the adverse impact of the global food production chain on human and planetary health [72].

In the CKD context, it is essential to mention that UPF contains additives and preservatives that represent a hidden source of highly bioavailable potassium, phosphorus, and sodium, which can contribute to mineral, bone, and cardiovascular disorders [74].

Diet-induced hyperphosphatemia exhibits a substantial link with diminished kidney function, increased risks of cardiovascular and all-cause mortality, and premature aging in CKD [7]. Proposed mechanisms argue that hyperphosphatemia promotes cellular senescence by promoting endothelial dysfunction since high levels of phosphorus lead to reduced release of nitric oxide and intracellular calcium, increased protein kinase C-β_2_, and apoptosis, in addition to fostering inhibitory phosphorylation of endothelial nitric oxide synthase [75]. Diets rich in fat and saturated fatty acids and low consumption of fruits, vegetables, and whole grains may also favor the development of intestinal dysbiosis in CKD, increasing proteolytic activities and favoring protein fermentation [73].

Additionally, red meat contributes to a higher protein, saturated fat, cholesterol, and iron intake, and an increased acid load. Previous studies have also shown that it can elevate the production of uremic toxins by the intestinal microbiota, which is linked to the development and progression of renal dysfunction and an increased risk of cardiovascular mortality [76]. Saturated fatty acids and cholesterol are associated with inflammation and increased cardiovascular risk in CKD, characterized by hypertriglyceridemia and low high-density lipoprotein (HDL) cholesterol [77]. Thus, red and processed meat is associated with a higher CKD risk [77].

High salt intake contributes to hypertension and exacerbates kidney damage—two interrelated conditions. Potential pathogenic mechanisms involve fluid overload, endothelial dysfunction, stimulation of the synthesis of pro-inflammatory cytokines, over-activation of sympathetic activity, and intrarenal production of angiotensin-II [78].

Sugar-sweetened beverages contain free sugars that cause a high glycemic load, contributing to metabolic issues like glucose intolerance, insulin resistance, and weight gain. In addition, it can lead to glomerular hyperfiltration and accelerate kidney function decline [79]. High-fat and high-sugar diets contribute to high energy intake, leading to a positive energy balance, fat accumulation, and obesity. Excess body fat, especially ectopic fat, interferes with normal metabolic functions and triggers inflammation. Specifically, intrarenal fat accumulation causes toxicity, and hemodynamic changes that result from obesity, such as hyperfiltration, albuminuria, and impaired eGFR, contribute to the appearance and progression of CKD [80].

**Figure 2:**
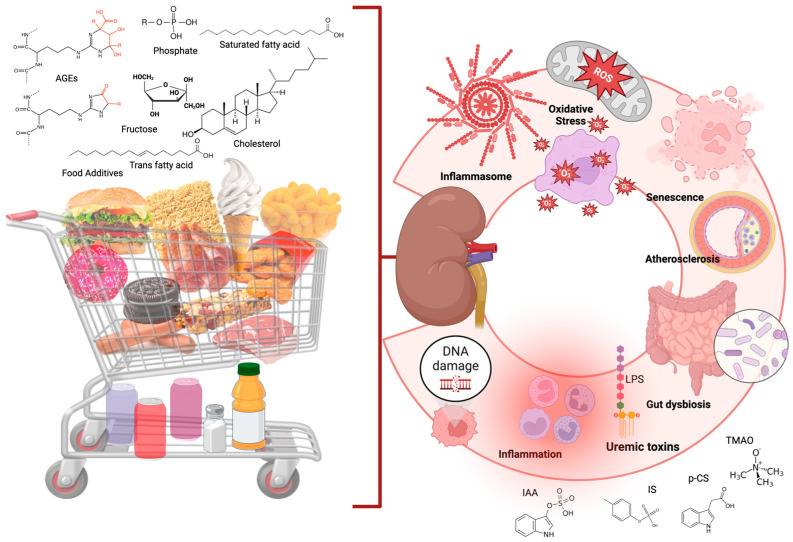
Effects of an unhealthy diet on kidney health. An unhealthy diet providing advanced glycation end-products (AGEs), cholesterol, salt, sugar, trans and saturated fatty acids, additives, and phosphate load induces oxidative stress, gut dysbiosis, and inflammation, harming the kidneys. Created by Biorender.com.

An unhealthy diet (providing AGEs, trans and saturated fatty acids, and additives) induces inflammation by activating inflammatory pathways (Figure 2), such as the NF-κB pathway [81]. Indeed, there is an overexpression of NF-κB and a consequent increase in pro-inflammatory cytokines in patients with CKD [82]. PYD domain-containing protein 3 (NLRP3) has also been identified as a promising candidate for mediating the inflammatory response in CKD through caspase 1, leading to the secretion of interleukin (IL)-1β and IL-18 and cellular pyroptosis [83].

In addition, addictive behaviors such as alcohol consumption may be associated with the development of CKD [84]. Public bodies consider alcohol consumption a risk factor for the global burden of disease, and it has been predicted that per capita consumption may reach up to 7.6 L of alcohol by 2030 [85]. The linear relationship between alcohol consumption and other chronic diseases, such as liver disease, CVD, obesity, metabolic syndrome, diabetes, various types of cancer, hemorrhagic stroke, or heart failure, is well covered in the literature. Such factors can directly and indirectly lead to CKD development [84]. This underscores the recommendation from public health organizations to limit alcohol consumption to minimize adverse health effects. Furthermore, the specific impact of alcohol on kidney health can vary depending on individual health conditions and drinking habits.

#### 2.2.2. Physical Inactivity

Physical inactivity is a modifiable risk factor for premature death and several non-communicable diseases, including CKD [86]. Recently, studies have demonstrated the role of physical activity in reducing the risk of developing CKD and limiting disease progression [87]. Using Mendelian randomization analysis, an observational cohort study investigated the causal relationship between physical activity and the impacts of certain sedentary behaviors on renal function in the population. Moderate to vigorous physical activity, as reported by the participants or measured by an accelerometer, correlated with a higher eGFR. Conversely, increased television viewing time was associated with a decreased eGFR and a higher prevalence of CKD [87].

Guo et al. explored relationships between regular physical activity and annual changes in the eGFR among approximately 200,000 adult participants from Taiwan. This study suggested that higher levels of habitual physical activity are associated with a smaller decline in the eGFR and a reduced risk of developing CKD [88]. Moeinzadeh et al. examined physical activity in 3374 Iranian adults with CKD. They found that a lack of physical activity increases the risk of developing CKD in its early stages. Furthermore, the authors concluded that physical activity could be recommended to reduce the progression of the disease and its associated impact. In another study involving 475,376 adults, physical activities, including those performed outside of work and ranging from low to moderate to vigorous intensity, were inversely associated with CKD risk [89]. This association encompassed conditions such as diabetic kidney disease, hypertensive nephropathy, and other forms of CKD [90].

A comprehensive systematic review and meta-analysis of observational cohort studies involving 1,281,727 general population participants showed that individuals engaging in higher levels of physical activity have a reduced risk of developing CKD compared to those with lower or no physical activity [91]. Additionally, evidence suggests increased physical activity levels are linked to metabolic health improvements in non-dialysis patients, such as lower serum triglyceride levels, reduced fat mass, and enhanced insulin sensitivity. These improvements may lead to a slower progression of CKD [92].

#### 2.2.3. Smoking

The relationship between smoking and health is widely explored in the literature. Kelly et al., in their systematic review evaluating modifiable lifestyle factors for the prevention of CKD, observed that smokers or ex-smokers were more likely to develop CKD compared to individuals who had never smoked [93]. In addition, an increased risk of needing renal replacement therapy was observed among current and ex-smokers [94]. Corroborating these results, Lee et al. observed that an extended period of smoking is positively associated with a higher risk of CKD progression and a greater need for renal replacement therapy [95].

It is known that CKD is accompanied by a burden of concomitant disease, such as the presence of hypertension, diabetes mellitus, dyslipidemia, and obesity, which are associated with a high risk of mortality, mainly from cardiovascular causes. Associated with this, smoking alone represents one of the leading causes of preventable death from CVD and cancer [96]. While smoking is known to impact kidney function adversely, the underlying mechanisms are still not fully understood [97]. Histopathological differences were observed in smokers, who presented increased myointimal hyperplasia of small arteries [98]. Furthermore, non-hemodynamic changes that cause kidney damage and reduced glomerular filtration can also be caused by oxidative stress, inflammation, decreased bioavailability of nitric oxide, increased concentration of endothelin-1, and increased secretion of vasopressin [99]. Smoking leads to transient and persistent increases in blood pressure, which have been associated with the progression of CKD [100].

On the other hand, smoking cessation is beneficial for kidney patients. Studies show that the longer the period of smoking cessation, the more significant the reduction in the risk of incident CVD [101]. Lee et al. showed that the risk of adverse renal outcomes decreases with more extended periods without smoking [95]. Thus, it is clear that smoking is a modifiable risk factor for health, and its cessation is a therapeutic target for public policies.

#### 2.2.4. Psychosocial Factors

Recent studies have investigated the association between mental health, especially in the context of anxiety and depression, and its influence on the development of CKD [102,103]. The mechanisms themselves still need to be explored in depth. An indirect link between mental health and the kidney–gut axis has been suggested, where the mental health score significantly mediated 9% of the association of functional gastrointestinal disorders with incident CKD [104].

A study involving 9313 participants with diabetes showed that the incidence of CKD over 5.7 years was associated with cases of depression and anxiety [105]. A cohort study from the Coronary Artery Risk Development in Young Adults study observed that CKD patients were significantly associated with a 36% higher risk of depression compared with non-CKD patients [103]. In this sense, it can be said that the association between mental health and CKD is bidirectional, where depression and anxiety can be risk factors for CKD, and CKD can be a cause of depression and anxiety [103].

Furthermore, mental stress is another crucial psychosocial factor that arises from psychological, physiological, or behavioral responses. Stressful factors can lead to physiological or emotional arousal, which affects the physical and mental health of individuals, with implications for the development and progression of CKD [106]. Stress can affect blood pressure and heart rate, reshaping the vascular reactivity of individuals, along with alterations in the activity of the sympathetic nervous system, the hypothalamic–pituitary–adrenal axis, and inflammatory cytokines [107]. These changes are closely linked to the pathophysiology of CKD, as the sympathetic system innervates all renal segments, with neural mechanisms also regulating sodium and water retention [106]. A recent large-scale study using data from 440,093 UK Biobank participants followed over 10 years demonstrated a significant association between stress-related psychiatric disorders and the development of CKD. The findings suggest that individuals with such disorders have an increased risk of developing CKD, even after accounting for genetic susceptibility, highlighting the potential role of psychosocial stress as a modifiable risk factor in kidney health [108].

Another critical point is the relationship between the higher incidence of CKD in individuals with serious mental illnesses, such as schizophrenia, which can be explained by the use of lithium treatment and more CVD [102]. Furthermore, individuals with serious mental illnesses receive suboptimal renal care, have fewer visits to nephrologists, and are less likely to receive a kidney transplant. It is suggested that care for these patients could be improved by educating the renal health team about the needs of patients with mental illness and facilitating closer collaboration with psychiatry [102,109]. Understanding how socioeconomic and psychosocial factors interact with kidney health may be essential to promoting equitable and prosperous interventions [110].

#### 2.2.5. Sleep Disorders

Sleep disorders like insomnia and sleep apnea syndrome are highly prevalent among patients with CKD, significantly impacting their quality of life and being linked to an elevated risk of cardiovascular events, such as stroke and myocardial infarction mortality [111,112,113]. Insomnia is a common condition, particularly among dialysis patients. Increased sleep latency and sleep fragmentation have been noted, attributed to poor sleep habits and frequent naps during dialysis, along with uremia itself, medications, and mood disorders such as anxiety and depression [114].

Regarding sleep apnea, the primary factors contributing to its development in patients with CKD are uremic neuropathy and myopathy, altered chemosensitivity, and hypervolemia [115]. On the other hand, sleep apnea syndrome can also be a risk factor for the development of CKD, since the hypoxia generated activates the sympathetic nervous system and can contribute to hypertension and increased oxidative stress in the renal tubules, having deleterious effects on renal function [116].

A systematic literature review of 44 articles (comprising 223,967 participants) revealed that the prevalence of sleep apnea in patients with CKD was 39.3%, with the highest rates observed in elderly patients. The study also indicated a 26.5% increase in mortality risk for individuals with sleep apnea compared to those without the comorbidity [117]. Furthermore, the cross-sectional and Mendelian randomization study showed that individuals with obstructive sleep apnea had a higher risk of CKD, with hypertension and obesity being conditions that increased this effect by 41.83% and 30.74%, respectively [118]. Another study also showed that in patients with CKD, the likelihood of obstructive sleep apnea is higher in obese and hypertensive males, with their quality of life being worse than that of those without obstructive sleep apnea [119].

Interestingly, an emerging area of research is the connection between obstructive sleep apnea, CKD, and the role of gut microbiome-derived toxins, particularly indoxyl sulfate. Hypoxia caused by sleep apnea impacts the gut microbiota composition and its permeability. Thus, these changes lead to an increase in bacterial species that produce indole from tryptophan. Disruption of the intestinal barrier elevates indole in the systemic circulation, increasing hepatic conversion to indoxyl sulfate. Furthermore, systemic inflammation, sympathetic hyperactivity, and oxidative stress induced by obstructive sleep apnea worsen the deleterious effects of indoxyl sulfate on renal tissue [120]. Therefore, the need to care for sleep quality in preventing and treating CKD is evident.

## 3. Therapeutic and Preventive Approaches Based on Exposome

With advances in exposome studies, a deeper understanding of the interaction between environmental factors and CKD promises to lead to more impactful and targeted prevention and control strategies. In this sense, green spaces have been recognized as an essential strategy from the point of view of kidney disease, promoting increased physical activity and social engagement, which are factors related to the prevention of non-communicable diseases, including diabetes, hypertension, and obesity, risk factors for CKD [121]. Green spaces can also contribute to reducing noise, extreme temperatures, and exposure to air pollution. A recent study with 346,697 participants without CKD followed for 12 years showed that green spaces can be protective factors against the development of CKD due to lower exposure to PM2.5 [122]. Similar findings were reported by Wang et al. [123], who demonstrated that the incidence of kidney diseases can be reduced through urban planning that incorporates more green spaces, particularly trees, within communities. This approach is especially beneficial for socioeconomically disadvantaged populations. Another study indicated that increased greenness was associated with a reduced risk of all-cause mortality in CKD patients and a slower disease progression [124].

Adopting a therapeutic approach focused on a balanced diet is essential in the context of therapies and preventive measures for CKD related to the exposome. This includes incorporating foods rich in bioactive compounds with antioxidant and anti-inflammatory benefits, such as curcumin, sulforaphane, propolis compounds, resveratrol, resistant starch, cinnamic acid, cinnamaldehyde, and allicin [73,83,125,126]. Moreover, decreasing the intake of UPF, which is high in chemical additives, helps reduce the body’s toxic burden, supporting effective preventive strategies for better health outcomes [72].

Plant-based diets have also been shown to be a safe alternative for preventing and treating CKD, from the perspective of green nephrology, allowing for a more significant reduction in protein intake than omnivorous diets. Furthermore, plant-based diets can reduce the environmental impact caused by raising large animals for food, reducing carbon footprint production, encouraging family and local agriculture, and using less water and soil [127]. Promoting plant-based diets to prevent CKD can decrease reliance on dialysis, reduce plastic waste, lower water usage, and decrease energy consumption. Ironically, the healthcare sector contributes to environmental health and climate change challenges, exacerbating resource depletion and greenhouse gas emissions [128].

In addition, with rising temperatures worldwide, it is critical to develop effective hydration plans and mitigate the adverse effects of prolonged heat exposure, such as patient education on prevention measures [129]. Patients should recognize signs of heat stress and stroke, monitor blood pressure and blood volume, and reassess patient prescriptions during heat waves [130]. Implementing comprehensive public policies addressing global warming and exposure to pesticides and heavy metals is an essential protective factor in preventing CKD [69]. This integrated approach contributes not only to the preservation of kidney health but also to the promotion of the population’s overall well-being.

Furthermore, the control of psychosocial factors that can trigger depression, anxiety, and stress and worsen sleep quality should also be mitigated through monitoring and investigation of these patients and a multidisciplinary approach with psychological and drug therapy, when necessary [131]. For example, adopting stress reduction programs based on mindfulness or cognitive–behavioral therapy can benefit CKD patients’ mental health [132,133]. The patient should also be informed about the importance of sleep quality, and recommendations to improve it are necessary, along with professional help to mitigate social, psychological, or pathophysiological problems that prevent them from having good quality sleep [134].

## 4. Concluding Remarks and Future Directions

**Figure 3:**
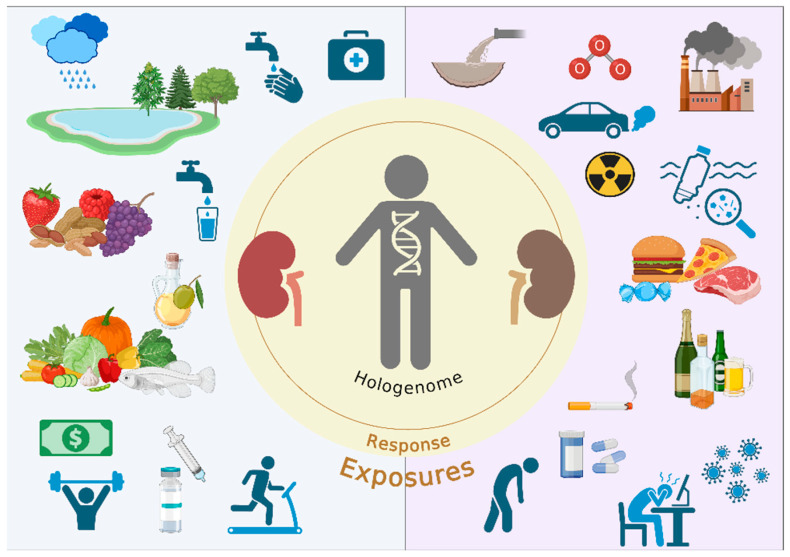
Exposome and its influence on renal health. Environmental exposures significantly influence the development of CKD. Climate change can worsen the incidence and prevalence of the disease, and food and its production processes contribute to the environmental impact. Urban living facilitates increased interaction among individuals and communities, exposing them to CKD-related environmental risk factors. Public policies encouraging green spaces, natural nutrition, and reducing gas and other pollutant emissions are essential for preventing CKD. Created by Biorender.com.

Environmental exposures have a significant influence on the development of CKD (Figure 3). Climate change, caused by an increase in atmospheric carbon dioxide and other greenhouse gases, can worsen the incidence and prevalence of the disease, as it generates significant cellular changes, culminating in changes in kidney function. Furthermore, food and its production processes contribute to environmental impact, mainly by promoting diets rich in ultra-processed products at the expense of natural foods. Urban living facilitates increased interaction among individuals and communities, exposing them to CKD-related environmental risk factors.

Exposome studies related to CKD offer a deeper examination of environmental exposures and create avenues for more effective prevention and treatment strategies. Recognizing the diverse range of exposomes contributing to CKD emphasizes adopting a holistic approach to renal health. Public health initiatives, policies to reduce harmful exposures, and individual lifestyle changes can help mitigate these risks and promote long-term health.

## Abbreviations

The following abbreviations are used in this manuscript:

CKD	Chronic kidney disease
CVD	Cardiovascular disease
AKI	Acute kidney injury
eGFR	Estimated glomerular filtration rate
NFκB	Nuclear factor-kappa B
PM	Particulate matter
ROS	Reactive oxygen species
ESKD	End-stage kidney disease
TIMP3	Metalloproteinase 3
Hg	Mercury
TCE	Trichloroethylene
PCE	Perchloroethylene
GSH	Glutathione
HCH	Hexachlorocyclohexane
GPSH	Glyphosate surfactant herbicide
NMDARs	N-methyl-D-aspartate receptors
Ca^2+^	Calcium ions
UPFs	Ultra-processed foods
HDL	High-density lipoprotein
NLRP3	PYD domain-containing protein 3
IL	Interleukin
